# Environmental Trigger(s) of Type 1 Diabetes: Why Is It So Difficult to Identify?

**DOI:** 10.1155/2015/847906

**Published:** 2015-03-30

**Authors:** Kjersti S. Rønningen, Jill M. Norris, Mikael Knip

**Affiliations:** ^1^Rikshospitalet, Oslo University Hospital, Oslo, Norway; ^2^Colorado School of Public Health, Anschutz Medical Campus, Aurora, CO, USA; ^3^Children's Hospital, Helsinki University Central Hospital, University of Helsinki, Helsinki, Finland

Type 1 diabetes (T1D) is one of the most common chronic diseases with childhood onset, and the disease incidence has increased from two- to fivefold over the past half century from, as of yet, unknown reasons. T1D occurs when the body's immune system turns against itself, destroying in a very specific and targeted way the pancreatic beta cells. T1D results from poorly defined interactions between susceptibility genes and environmental determinants. In contrast to the rapid progress in finding T1D genes, the identification and confirmation of environmental trigger(s) remain a formidable challenge. The identification of environmental determinant(s) responsible for the development of or protection against T1D is crucial from that point of view that they may be modifiable and might ultimately be used in disease prevention. The high costs of the disease both to the society and to the affected individuals and their families imply that preventing a fraction of cases, or even delaying the onset, will be of high value.

This special issue contains original research articles as well as review articles that could stimulate the continuing efforts in the identification of exogenous triggers in the development of T1D. New data on environmental factors associated with progression to clinical T1D was of particular interest. Articles using bioinformatics to evaluate the number of cases (either autoantibody positivity or clinically recognized disease as the case definition) needed for the identification of a certain environmental trigger were as well very interesting. In addition, this special issue includes articles presenting autoantibody patterns in the prediction of T1D and cellular and molecular mechanisms in the disease pathogenesis. Here we give a short overview of the content of the current special issue.

In original research articles, the hygiene hypothesis and risk for T1D were studied prospectively in the Diabetes Autoimmunity Study in the Young (DAISY) by examining daycare attendance during the first 2 years of life. Altogether 1783 children were available with complete daycare and breastfeeding data. For the first time, the data obtained suggest that breastfeeding may modify the effect of daycare on the risk of T1D.

The goal of the other DAISY study included was to demonstrate methods for identifying exposures that differentially influence the disease process at certain ages by testing for age-related heterogeneity. The DAISY study has followed 2547 children at increased risk for T1D from birth, and 188 have developed islet autoimmunity (IA). Ethnicity, maternal age, and erythrocyte membrane n-3 fatty acid levels demonstrated significant age-related associations with IA risk in children at increased risk of T1D. For example, increased n-3 fatty acid levels were associated with a significantly decreased risk of IA after the age of 4.25 years, but not before, which may have implications in the design of intervention studies.

In a study using carbohydrate counting in 22 cases compared to 15 controls on standard diabetic diet, it was shown that carbohydrate counting may be a functional method in patients affected by T1D.

In review articles, the role of maternal dietary essential fatty acids (EFA) was assessed in a review of studies on nonobese diabetic (NOD) mice. The paper concluded that, among maternal factors, dietary n-6/n-3 EFA ratio during pregnancy and the lactation period may affect the severity of insulitis and the progression to autoimmune diabetes in the offspring.

In a review on Luminex and other multiplex high throughput technologies for the identification of environmental triggers of T1D, critical issues are discussed and potential solutions are offered for the development of optimal assays. Based on their own experiences, the authors conclude that multiplex technologies might be successfully used for the evaluation of different classes of environmental exposures and host responses in T1D pathogenesis.

Another review summarizes the association between environmental chemical exposure and T1D development. Chemicals may have direct toxic effects on insulin producing beta cells, they may have immunomodulatory effects, and/or they alter hormone levels affecting gut microbiota or intestinal permeability. Due to the lack of strong evidence for a single factor as the major trigger of T1D, it is tempting to propose that several factors have additive or synergistic effects, acting via several mechanisms and/or at different stages in the disease process.

One of the review articles focused on factors which have to be evaluated and decisions to be taken before starting a new prospective cohort study. Before the recruitment starts, it is essential to design the study in an optimal way to be able to identify important environmental factors. The paper titled “Environmental Trigger(s) of Type 1 Diabetes: Why So Difficult to Identify?” has been used for the discussion of Dr. Rønningen's own experiences. Most likely there are already sufficient prospective cohort data collected or under collection to identify the environmental trigger(s) of T1D. New valuable information on exogenous factors and their contribution associated with the development of beta cell autoimmunity and progression to T1D could be achieved by a huge international collaborative effort. Such an effort would make it possible to integrate demographic, genetic, autoimmune, and exposure data from the existing cohorts in Finland, Norway, Sweden, Germany, and Denver, Colorado, in addition to the TEDDY study generating comprehensive information on the role of environmental factors in the development of T1D both in a family cohort and in a population-based cohort.

*Kjersti S. Rønningen* *Jill M. Norris* *Mikael Knip*

